# Evaluation of an Evidence-Based Weight Loss Trial for Urban African American Adolescents and Caregivers

**Published:** 2017-09-11

**Authors:** K Campbell-Voytal, KB Hartlieb, PB Cunningham, AJ Jacques-Tiura, DA Ellis, K LC Jen, S Naar

**Affiliations:** 1 Family Medicine & Public Health Sciences, Wayne State University School of Medicine, USA; 2 Department of Humanities, Health & Society, Herbert Wertheim College of Medicine, Florida International University, USA; 3 Department of Psychiatry & Behavioral Sciences, Medical University of South Carolina, USA; 4 Nutrition and Food Science, Wayne State University, USA

**Keywords:** Adolescence, Family, Qualitative methods, African-americans [u.s.], Program evaluation, Weight loss

## Abstract

Rates of obesity are among the highest for African American adolescents in the US. However, African American adolescents benefit the least from evidence-based weight loss interventions, often experiencing poor treatment retention and low motivation. Participant evaluations provide key information for future development of family-based weight loss interventions able to address these barriers. The purpose of this study was to examine the experiences of African American adolescent and caregivers participating in the FIT Families trial for program satisfaction and content palatability. Content analysis was used to analyze semi-structured exit interviews from 136 African American adolescents [median age 14 years, 69% female] and caregiver pairs [primarily mothers] participating in a family-based 6-month behavioral weight loss intervention that was delivered either in the home or in an office setting. Participants reported most program practices [location, parent involvement, interpersonal relationship with weight loss counselors] and intervention content [cognitive behavioral skills training, motivational interviewing, contingency management] were helpful. Many adolescents [49%] and their caregivers [47%] reported that the program was acceptable overall, however noted that areas for refinement did exist. Participants reported that managing the logistics of weekly sessions was hard. Families expressed a desire for more engaging skills-based learning and the inclusion of exercise sessions and additional tailoring to needs and interests. Individualization, active learning, and support around parenting continues to be beneficial when designing interventions.

## Introduction

Rates of obesity are alarmingly high among ethnic minorities, particularly African American [AA] adolescents [1]. Obesity prevalence rates for AA female adolescents, which exceed 24.4%, are the highest for any demographic group [1]. Obesity is a major public health concern that requires effective intervention if these trends and associated comorbidities [e.g., hypertension, cardiovascular disease, type 2 diabetes] are to be reduced. Unfortunately, effective weight loss interventions for AA adolescents are rare [2-8].

Leading academics and professional organizations committed to reducing obesity and health disparities in AA youth have strongly recommend the use of behavioral interventions to increase healthy eating and activity level [9]. While effective weight loss strategies for the general population are well known, behavioral interventions to increase healthy eating and activity among AA adolescents have largely failed [10,11]. Almost every major randomized clinical trial designed to reduce obesity or overweight among African American or other ethnic minority adolescents failed to produce significant weight loss [4,5,7,9].

Two of the most prominent factors that may account for these disappointing results with AA adolescents include poor treatment retention and low motivation [3,8,12,13]. These two factors along with the urgent need to construct effective weight loss interventions for AA adolescents served as the impetus for the FIT Families intervention [14]. FIT Families tested home and community based delivery of the weight loss content in order to address barriers to program attendance using a 6-month, Sequential Multiple Assignment Randomized Trial [SMART] [15]. The intervention delivered evidence-based cognitive behavioral weight loss skills, such as monitoring food intake and activity level, environmental control to reduce cues to eat unhealthy foods, and goal setting, plus integrated Motivational Interviewing [MI-CBS] to all families. Motivational interviewing focuses on the compassionate elicitation and strengthening of intrinsic motivation for behavior change [16]. In addition, some families were randomized to receive Contingency Management [CM], extrinsic motivational content using operant conditioning principles to encourage completion of behavioral goals to address motivational barriers to weight loss [17-19]. At baseline families were randomized to receive MI-CBS either in the home or the office. After 3 months, participants who did not meet a 3% weight loss target were re-randomized to receive twice weekly, home-based sessions involving either CM or additional MI-CBS. Families of adolescents who met the 3% weight loss goal were assigned to a single weekly maintenance session.

FIT Families produced promising outcomes in terms of engaging minority families in treatment, weight loss for subsets of adolescents, and improvements in health indices [14]. The modest intervention effects on adolescent weight outcomes, however, suggest that additional efforts are needed to enhance weight loss in ethnic minority populations. A qualitative framework allows researchers to understand the perspectives of participants around process phenomena that are not easily measured [20]. Qualitative methodologies are particularly well suited for inquiries into the experiences of adolescent participants yet the literature is limited in the area of minority weight loss intervention feedback [21]. Thus, the current study used qualitative methods to examine the experiences of both adolescent and caregiver FIT Families participants in the areas of program content and delivery.

## Methods

### Study setting and population

The FIT Families trial was implemented from 2011 to 2013 in a large, U.S. Midwestern city. Caregivers and adolescents were recruited through pediatric clinics in a large urban teaching hospital and the community [22]. Adolescents were eligible if they self-identified as African American, were age 12-16 years and had a Body Mass Index [BMI] at the 95th percentile or more for their age and gender. Caregivers were eligible if they were age 18 years or older, a legal guardian [or had consent of the guardian], resided within 30 miles of the study offices, and were willing to participate with their adolescent. Research protocols were approved by the Wayne State University Institutional Review Board.

### Procedures

**Figure 1:**
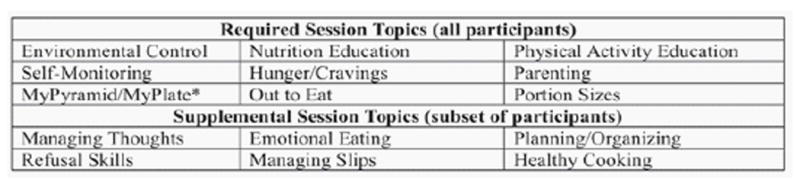
List of required and supplemental session topics. *Educational content was provided to families on the USDA My Pyramid until the 2011 debut of the USDA My Plate.

All FIT Families participants were contacted by research assistants within 1 month of treatment completion to participate in exit interviews. Exit interviews were conducted using a semi-structured interview guide developed to explore participants’ experiences in the following areas; 1] overall FIT Families program likes/dislikes and areas for improvements/change; 2] specific evidence-based session topics that were helpful/unhelpful as well as content areas that could be added, and 3] the impact of intrinsic and extrinsic motivational strategies (Figure 1). Participants were also asked to reflect feelings about working together [adolescent and caregiver] throughout the program. Interviews were conducted with the adolescent and caregiver separately in their home.

Research interviewers were trained in two cohorts using a semi-structured interview format. Three interviewers conducted all interviews; two were male, two were African American, and one was Caucasian. Training included two workshops reviewing the protocol and interviewing technique, practice, and a final demonstration using a proxy adolescent or caregiver reviewed by the authors. Interviewers were required to demonstrate fidelity to the interview protocol before completing a formal interview.

Exit interviews were completed by 75% of study participants [136 adolescent-caregiver dyads]. Participants answered interview questions based on the randomization received [home vs. office; CM *vs*. additional MI-CBS]. Adolescent participants were primarily female [69%], median age 14 years, with an average BMI percentile 98.9 at baseline. Caregivers were predominantly mothers [89.7%], mean age 43.3 years, employed in the home [52%], with an average of 2.1 other minor children present. Families participating in the exit interviews were not significantly different from non-participants, except that they had more minor children in the home [M=2.07, SD=1.19] compared to nonparticipants [M=1.58, SD=.97]; t[179] = 2.53, p= .012. Demographic characteristics of participants are summarized in (Table 1).

### Analyses

All interviews were audio recorded and professionally transcribed verbatim. Transcripts were entered and coded using NVivo 10 Qualitative Software [QSR International, Melbourne, Australia]. Individual code lists for adolescents and caregivers were developed by the research team through iterative series of co-coding and discussions of a random set of transcripts from five dyads. Major code categories were structured by interview question and sub codes by response content. All transcripts were coded by a single coder and 20% transcripts were randomly selected for dual coding to monitor coding reliability. Differences were discussed and reconciled by consensus. Coding differences not resolved were dropped. For this study, we summarize results by helpfulness or unhelpfulness of session content, program likes and dislikes, and impressions of intrinsic and extrinsic motivational components. Additionally, in order to inform intervention improvement, we present a summary of overarching participant recommendations.

## Results

### Helpful session content

Adolescent and caregiver participants overwhelmingly agreed that most of the evidence-based session topics were helpful, and had similar endorsements of topics felt to be particularly helpful [see Table 2-Session Topic Preferences]. For example, both adolescents [n=59, 43%] and caregivers [n=67, 49%] found portion size to be the most helpful topic, followed by “managing cravings” and “environmental control” as topics most helpful to adolescents. Caregivers ranked nutrition education [registered dietitian-led content including energy balance and label reading] and healthy cooking sessions as second and third most valuable sessions.

Responses to probes about “how the content helped” clustered into two areas. In the first area, youth and caregivers expressed that the session “increased awareness”. These responses centered on expanded understanding or new learning. Youth: “Cause I knew how much I was supposed to have on my plate then. And I really didn’t know that” [345].

The second area, “applied a new technique”, captured concrete examples of youth and caregivers using new strategies in daily life. Caregiver: “My kids think your plate is supposed to be full. When you show them the portion sizes on the little plate, it’s like ‘You’re eating too much’. I actually downsized my plates too” [334].

### Unhelpful Session Content

Session content covered in “out to eat” was the session most often identified as unhelpful by both adolescents [n=20, 15%] and caregivers [n=29, 21%]. Similarly, some adolescents [n=21, 15%] and caregivers [n=13, 10%] found that “managing cravings” session content was unhelpful. Summarizes these findings Table 2.

When asked how the session content was unhelpful, adolescents and caregivers stated that the content “did not work for me/my child”. Adolescents’ [n=63, 56%] expressed that either they didn’t need the content [“I don’t go into cravings”339] or that it was too difficult to implement, [“If I had to be somewhere or somebody else made my plate, or just eating out, it’s kind of hard to stop and sit and look, okay, do I need this much?”357]. Caregivers [n=79, 79%] also expressed that either the intervention content didn’t fit the family lifestyle [“It was hard to just plan a meal when you get home at six or seven. I think that was the hardest thing, planning a meal.”307] or those they felt that their adolescent did not benefit from the strategy [“…the log was difficult to keep on track …because a lot of time she would forget…”365].

### Program structure

The logistics of scheduling, traveling for some of the office-based families, and coordinating meeting times around the school day and afterschool activities of youth, and work schedules of caregivers was challenging for some families.

**Caregiver**: “It’s that it went into my work schedule, my personal schedule. When on the day, I had to look out how it benefited my son, and had to put that all aside. But a lot of days I was rushing home from work or sometimes I didn’t make the sessions. That’s what I didn’t like about it. But it wasn’t directly anybody’s fault. It’s just my situation” [349].

Families also found the sessions redundant. Adolescent “I think it was just because we talked about the same thing over and over again, which took away my interest”[381].

Regarding the frequency of sessions, the vast majority of caregivers and adolescents felt that the twice weekly session frequency and duration were “just right”. There was a notable subgroup, though, that felt that sessions lasting 60-90 minutes were too long.

**Caregiver**: “Anything over an hour that was just way too much” [380].

**Adolescent**: “Forty-five minutes [would be better] ‘cuz after 45 minutes people tend to lose their concentration on things” [306].

Dislikes with office location for caregivers were related to difficulty in transportation and travel time. There were also complaints about the cab company and their drivers that provided transportation for families without transportation. Dislikes with home location for both teens and caregivers were related to preferring to avoid the distractions of home; beliefs that the clinic would offer more privacy and that they could focus better on the session.

**Caregiver**: “If you’re at home, you’re comfortable, and you’re more distracted with a lot of different stuff going on with the other kids being around…If you were in a different setting you’re paying more attention and everything at home is not distracting you… I don’t have to …still playing mom to them all at the same time”[371].

Adolescent: “When I’m here, I’m at home I’m just like relaxing. So it was difficult to get into the right frame of mind and to focus” [436].

Families reported additional barriers to attendance. These included a wide variety of scheduling difficulties, with school activities mentioned most often. Illness of the caregivers, teens and other family members, and unanticipated emergencies [e.g. deaths and interpersonal conflict] were also barriers to attendance.

Finally, when questioned about their perceptions around working together [i.e., adolescent and caregiver] during the program, the majority of caregiver and youth responses were strongly positive. Caregivers valued joining with the youth to share mutual commitment to change. A subset of caregivers shared that there were limitations to involvement albeit to avoid conflict or the need for youth independence.

**Caregiver**: “I need y’all to take the lead because me and [adolescent], we’ve been through this, so she’s kind of used to me,’Wah, Wah, Wah’ in her ear. She’s not hearing me anymore” [423].

**Caregiver**: “I’m still just confused and kind of frustrated. I feel kind of lost, ‘cause I still don’t know what to do [controlling adolescent’s eating]” [423].

At the same time, some youth stated that more parental involvement would have improved the program. Adolescent: “…go out more [together], do more physical activities [with my mom]… like going for a walk or something” [340].

### Intrinsic and extrinsic motivation

FIT Families sought to increase motivation in two distinct ways: intrinsic motivation via motivational interviewing, as indexed by participants’ satisfaction with the Community Health Worker [CHW] and extrinsic motivation via contingency management. Overall, participants expressed strong satisfaction with their CHW, who were trained in MI. The strength of these comments affirms overall participant satisfaction with providers versed in MI principles. Families appreciated the non-judgmental, collaborative, and compassionate interaction style of the interventionists.

**Caregiver**: “She was very, very good, very, very professional in how she was able to redirect him [his resistance] and to bring him back in and help him to understand how the program is beneficial for him” [448].

Some caregivers, as a key source of support for adolescent weight loss, expressed difficulties with parenting and feeling the responsibility of keeping the youth motivated.

**Caregiver**: “For me the hardest part was keeping him motivated to do his logs. I was trying to keep positive. When he wouldn’t do the logs no matter how much I reminded him, it’s hard to keep positive” [338].

On the other hand, some caregivers also shared that over time they noticed the youth internalize the program and parenting got easier.

**Caregiver**: “…when she really got into it, really, really into it, that was the easiest part. I didn’t have to be on her…about what she eat and what she can’t eat…” [454].

Contingency Management [CM], the extrinsic motivation component of FIT, provided reinforcement for program attendance and weight loss. Approximately half of participants were assigned to the CM arm of treatment. Caregivers and youth were asked if CM affected their motivation for attendance and for completing weight loss goals. Overall, 73% of families endorsed that CM had a positive effect on the youth’s motivation or attendance.

**Youth**: “I already had motivation but, you know, I was getting points from that, I was like, “Oh yeah!.” I was a little too excited, but that called me to work out a lot harder… that boosted my motivation, yeah” [428].

## Discussion

FIT Families is a family-based intervention incorporating evidence-based cognitive behavioral and motivational strategies associated with effective behavior change. To our knowledge this is the first qualitative study targeting a large number of obese AA adolescents and their caregivers having participated in a randomized clinical trial. The obesity treatment field has a checkered history of effectively retaining, recruiting, and helping obese AA adolescents lose weight [2-8]. The results of the current study indicate that family- and evidence-based interventions are quite palatable for AA adolescents and their caregivers as suggested by the overwhelming endorsement of FIT Families session topics, program characteristics, and motivational strategies. Session topics [which were chosen because of their empirical support] such as environmental control, nutrition education [portion sizes, label reading], and physical activity education, were positively endorsed by adolescents and caregivers at similar rates. This finding alone should encourage obesity researchers to continue to use evidence-based behavioral strategies with AA adolescents, albeit with certain caveats.

**Figure 2:**
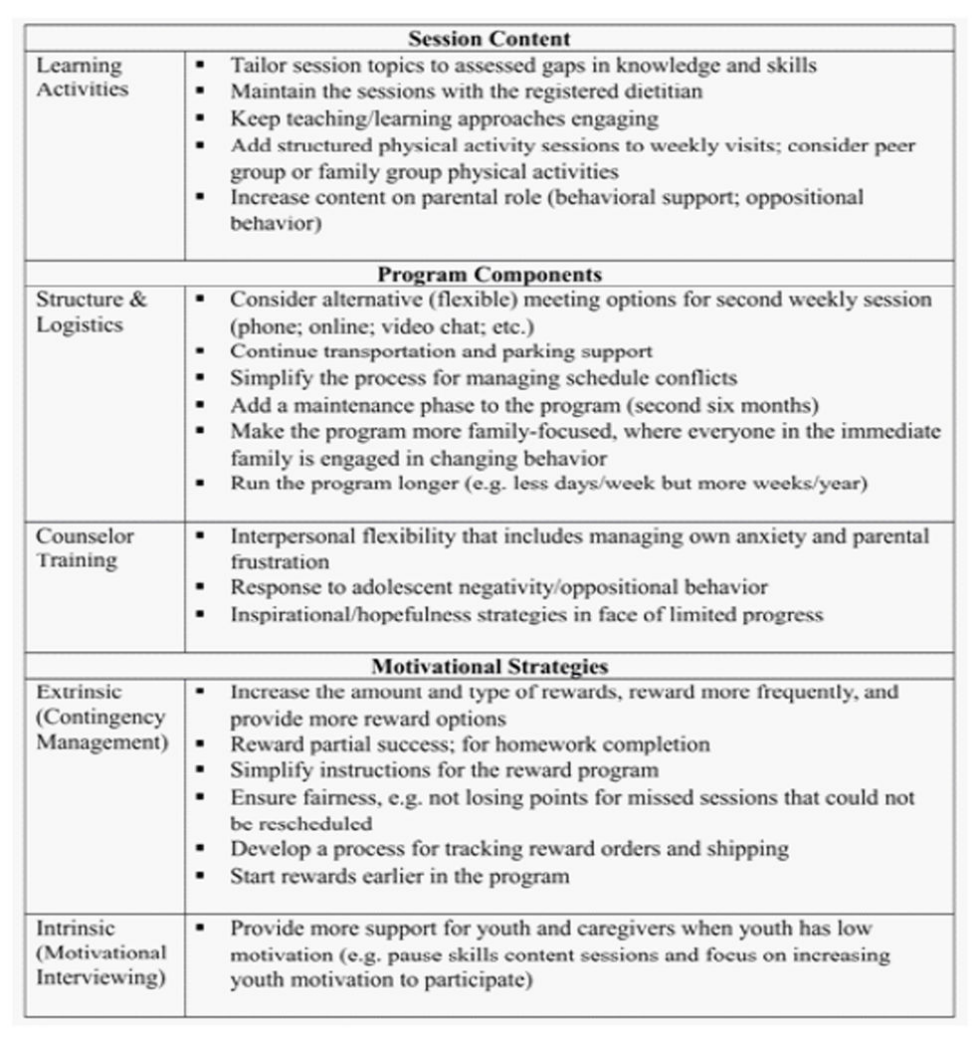
FIT Families. Feedback: Key recommendations.

The overarching recommendations shared by families fall into the areas of session content, program components, and intervention motivational strategies. Summarizes the feedback from FIT Families participants. Consistent with positively endorsed features of other weight management interventions adolescents and caregivers expressed a strong desire for sessions to include more active, non-didactic learning activities to keep adolescents engaged during sessions (Figure 2) [23]. Community Health Workers mechanically following session structure for specific content as required for internal validity in randomized clinical experiments came across as disengaging for some adolescents and caregivers. This was particularly evident in adolescent and caregiver comments suggesting that didactic instruction about physical activity and actual performance of physical activity are quite distinct. Adolescents and caregivers concurred that sessions that involved actual physical activity would benefit future programs.

Similarly, some FIT Families session topics and activities proved to be quite burdensome for a subset of adolescents and caregivers. This was especially true for self-monitoring of nutrition and physical activity, and having multiple sessions per week. For example, approximately 20% of adolescents found keeping a record of food and physical activity a burden, second only to diet management [e.g. giving up junk food]. A subset of families suggested that two sessions per week sessions were hard for busy families to manage and often were seen as redundant and or unnecessary. Similarly, travel time to clinic sessions was a heavy burden for families, especially those relying on public transportation. On the other hand, some families also expressed a desire for the sessions to include more family members to support behavior change. Taken together these comments suggest a need for flexibility in how information is presented during sessions, balancing didactic and active learning strategies, and finding ways to decrease program burden.

While there was minimal endorsement of unhelpful intervention content, what youth and caregivers did express suggests that future studies should specifically adapt intervention content to meet the unique strengths and needs of each family. For example, in this sample the content associated with eating out did not seem particularly helpful. Despite strong evidence that decreasing visits to restaurants and modifying meals to include more nutrient dense choices as healthy weight promoting, families commented on the inconvenience of bringing resources with them to assist with ordering, decreased satisfaction with the meal if it didn’t include the higher calorie option, and that the frequency of eating out practices was too low to have an impact on their child’s weight [24, 25]. When developing interventions, researchers are faced with the balance of packaging the intervention for reproducibility and consistency with the recommendations for individual optimization of content.

Behavior change is fraught with difficulty. However, most adolescents and caregivers found working together to be beneficial and enjoyable. Despite the potential for a struggle around independence in the adolescent years, the families found support and strength in the team approach. A 2015 review of the influence of parental participation on weight outcomes in African American adolescent females also concluded that strong parental involvement in obesity related interventions can facilitate weight loss and promote improved dietary intake and increased physical activity [26]. Furthermore, the incorporation of extrinsic motivation strategies [Contingency Management] benefits from parental involvement [19]. However, there was a subset of families that found the experience challenging at times, especially when there was minimal or no weight loss. Thus, it was not surprising that some caregivers suggested more help with parenting strategies to assist with motivating their child to participate. Some adolescents suggested that sessions be divided into sections with the caregiver/adolescent being seen alone first and then together, speaking again to the need for flexibility in intervention delivery. Parental involvement remains an important component of behavior change in minority families.

### Study limitations

Present study participants may have had a more positive experience in the intervention and therefore their perspectives may not reflect the views of those who chose not to participate. The gender distribution of adolescents in the exit interviews reflected the larger trial, however, adolescent boys and fathers were underrepresented which impacts generalizability. Gender-specific preferences and interactions may not be well represented in our findings. Finally, the purpose of the exit interview was to uncover perspectives and experiences during participation. The interview guide specifically addressed caregiver-adolescent interactions in intervention activities but did not specifically probe other important family conditions and social interactions that could contribute to weight loss outcomes [e.g. peer and other supportive relationships].

### Implications and contributions

There is little in the literature about the perspectives of African American adolescents and families enrolled in a weight-loss trial. This study reveals that a science-based nutrition and physical activity intervention delivered by CHWs trained in Motivational Interviewing and Contingency Management strategies was generally well received. However, African American families were less satisfied with the reliance on protocol-driven training and preferred greater choice over training session topics and active learning opportunities. Parents also requested help with effectively communicating with their adolescent about weight and behavior change. These suggestions are likely important components for developing culturally tailored weight loss interventions for African American youth and families in the future.

## Figures and Tables

**Table 1:** Participant characteristics.

Characteristic	Description	N (%)
Adolescent age	12	33 (24.3)
	13	33 (24.3)
	14	24 (17.6)
	15	28 (20.6)
	16	18 (13.2)
Adolescent age	Mean (SD)	13.74 (1.37)
Adolescent gender	Female	94 (69)
BMI Percentile	Mean (SD)	98.9 (1.02)
Caregiver role	Mother	122 (89.7)
	Father	5 (3.6)
	Grandmother	7 (5.1)
	Other female family member (sister, aunt)	2 (1.4)
Caregiver employed	No (%)	52
Minor Siblings in Home	Mean (SD)	2.07 (1.19)

**Table 2:** Teen & caregiver preferences for session topics.

Characteristic	Description	N (%)
Adolescent age	12	33 (24.3)
	13	33 (24.3)
	14	24 (17.6)
	15	28 (20.6)
	16	18 (13.2)
Adolescent age	Mean (SD)	13.74 (1.37)
Adolescent gender	Female	94 (69)
BMI Percentile	Mean (SD)	98.9 (1.02)
Caregiver role	Mother	122 (89.7)
	Father	5 (3.6)
	Grandmother	7 (5.1)
	Other female family member (sister, aunt)	2 (1.4)
Caregiver employed	No (%)	52
Minor Siblings in Home	Mean (SD)	2.07 (1.19)
